# Design of a Multiepitope Pan-Proteomic mRNA Vaccine Construct Against African Swine Fever Virus: A Reverse Vaccinology Approach

**DOI:** 10.1155/vmi/2638167

**Published:** 2025-01-04

**Authors:** Ella Mae Joy S. Sira, Lauren Emily Fajardo, Edward C. Banico, Nyzar Mabeth O. Odchimar, Fredmoore L. Orosco

**Affiliations:** ^1^Department of Science and Technology, Virology and Vaccine Research Program, Industrial Technology Development Institute, Bicutan, Taguig 1634, Philippines; ^2^Department of Biology, College of Arts and Sciences, University of the Philippines Manila, Manila 1000, Philippines; ^3^Department of Science and Technology, S&T Fellows Program, Bicutan, Taguig 1634, Philippines

**Keywords:** African swine fever, epitopes, mRNA vaccines, reverse vaccinology

## Abstract

African swine fever (ASF), caused by African swine fever virus (ASFV), is a highly contagious disease with devastating effects on the global pig industry. This warrants the development of effective control strategies, such as vaccines. However, previously developed inactivated vaccines have proven ineffective, while live-attenuated vaccines carry inherent safety risks. The use of mRNA vaccines eliminates these risks offering a safe, cost-effective, and efficient vaccine strategy against ASFV. In this study, a reverse vaccinology approach was used to design a multiepitope pan-proteomic mRNA vaccine against ASFV. Various bioinformatics tools were employed to predict epitopes for cytotoxic T lymphocytes, helper T lymphocytes, and B lymphocytes. A 50S ribosomal L7/L12 protein adjuvant, 5′ cap, poly(A) tail, signal peptide, and MHC-I-targeting domain were incorporated into the design using appropriate linkers to increase immunogenicity, stability, and recognition efficiency. The physicochemical properties of the final construct were evaluated, and docking analyses were done with Toll-like receptors (TLRs) 3, 4, and 7 to evaluate binding affinity. A molecular dynamics simulation was then performed to determine binding stability, while immune simulations evaluated host's immune response. Based on 100 ASFV proteomes, six epitopes that induce cytotoxic T-cell responses, five epitopes that induce helper T-cell responses, and four epitopes that induce antibody production were predicted. The designed vaccine construct was found to be nonallergenic, antigenic, and stable when bound to TLR4 while the binding pocket analyses of the vaccine construct to TLR3 and TLR7 indicate high translation efficiency. Immune simulations demonstrated successful induction of immune responses and generation of antigen-specific memory cells. In conclusion, this study introduces an mRNA vaccine construct as a potential disease control strategy against ASF for in vitro confirmation.

## 1. Introduction

African swine fever (ASF), caused by African swine fever virus (ASFV), is a highly contagious disease that affects both domestic pigs and wild boars. Pigs play a significant role in the livelihoods of rural and periurban populations in Southeast Asia, which accounts for over 60% of the world's domestic pig population [1]. Since January 2022 [2], ASFV has caused outbreaks in several regions amounting to a total loss of 1,406,064 pigs, with outbreaks outside Africa linked to ASFV Genotypes I and II [3, 4]. In the Philippines, ASFV outbreaks have led to the culling of over 300,000 pigs and a decrease in the growth rate of pork production by 20.8% in 2021, significantly affecting the Philippine pork industry [5]. With its high transmissibility, mortality, and morbidity rates of up to 100%, ASF remains a threat to the pig industry, impacting both the economy and biodiversity.

ASFV, an enveloped virus containing a double-stranded DNA of 170–194 kb encoding more than 160 genes, is the only member of the *Asfarviridae* family [6]. ASFV is comprised of a large DNA genome enclosed by a core shell, an inner lipid envelope, and an icosahedral capsid [7]. It mainly targets monocytes and macrophages in domestic and wild swine but may also replicate in hepatocytes, renal tubular epithelial cells, neutrophils, and endothelial cells [8].

Vaccination is one of the best strategies for controlling ASFV. Past efforts in the development of vaccines against ASF include inactivated vaccines, which have been proven ineffective, and live-attenuated vaccines, such as ASFV-G-ΔI177L—the first commercial ASF vaccine in the world, developed in Vietnam. However, live-attenuated vaccines carry inherent risks, such as reversion to the virulent phenotype and, in the case of DNA vaccines, possible host genome integration [9, 10]. The use of mRNA vaccines over conventional vaccines completely eliminates these risks, making it a safe, cost-effective, and efficient alternative.

mRNAs have been proven to promote both humoral and cellular immune responses. Furthermore, a change in the encoded antigen does not affect the physicochemical properties of the mRNA backbone, allowing for possible standardization of production [11]. Such instances include regulation of its half-life through various modifications and delivery methods, downmodulation of its inherent immunogenicity to increase its safety profile, and increased efficacy through several modifications to improve its translatability and stability. More importantly, since it is a minimal genetic vector, repeated administration of mRNA vaccines is not an issue; hence, antivector immunity is completely avoided [12].

Currently, the development of mRNA vaccines mainly focuses on the protection against zoonotic diseases, such as foot-and-mouth disease, rabies, influenza, and mosquito-borne flaviviruses. mRNA vaccines developed against these pathogens exhibited stronger immune responses with an evidently increased T-cell immune response as compared to their traditional vaccine counterparts [13]. Swine-specific custom prescription products using RNA technology have also been developed to target several viruses, including PCV2, PSV3, rotavirus, sapovirus, influenza A virus, and porcine epidemic diarrhea virus. These products by Merck were introduced in 2018 and are licensed for use in swine by USDA under the brand name Sequivity [14]. However, there are currently no RNA-based products available for ASFV.

Using genomic and proteomic data of the target pathogen, reverse vaccinology (RV) has accelerated the discovery and development of mRNA vaccines [15]. Thus, this study aimed to design a multiepitope pan-proteomic mRNA vaccine against ASF using an RV approach.

## 2. Methodology

### 2.1. Proteome Retrieval and Conservancy Analysis

The approach for multiepitope subunit vaccine design utilized in this study follows the methodology of Simbulan et al. [16] from the same research group. All available ASFV proteomes were retrieved from the National Center for Biotechnology Information (NCBI) (https://www.ncbi.nlm.nih.gov) and the Universal Protein Resource (UniProt) (https://www.uniprot.org/). The retrieved proteomes were aligned using MAFFT v7 (https://mafft.cbrc.jp/alignment/server/) with the default parameters. A neighbor-joining (NJ) tree was constructed using the same server under the JTR model with 1000 bootstrapping replications. ASFV genotypes were identified through clade clustering, using isolates with available NCBI genotype assignments as references. Representative proteins with > 80% sequence similarity and > 75% alignment coverage were identified by CD-hit clustering (https://sites.google.com/view/cd-hit). To address recent global ASFV outbreaks, proteins found in ≥ 80% of Genotype I or ≥ 80% of Genotype II were prioritized.

The clustered protein sequences were aligned using Clustal Omega (https://www.ebi.ac.uk/Tools/msa/clustalo) and uploaded to the Protein Variability Server (PVS) (https://imed.med.ucm.es/PVS) with a variability threshold of 1 [17, 18]. Variability-masked sequences were used for the linear B lymphocyte (LBL) epitope prediction. Consecutive residues forming fragments ≥ 9 residues long and ≥ 15 residues long were retained for cytotoxic T lymphocyte (CTL) epitope prediction and helper T lymphocyte (HTL) epitope prediction, respectively.

### 2.2. CTL Epitope Prediction

The binding affinity of potential epitopes to major histocompatibility complex Class I (MHC-I) was predicted using the immune epitope database analysis (IEDB) MHC-I binding prediction tool (https://tools.iedb.org/mhci), with epitopes having a percentile rank (PR) of ≤ 0.5 considered to be strong binders. Epitopes with strong binding affinities to at least 20% of the MHC molecules used were identified to be promiscuous and considered for further evaluation [19]. Promiscuous strong binding 9-mer peptides were evaluated for CTL epitope processing using NetCTLpan 1.1 (https://services.healthtech.dtu.dk/services/NetCTLpan-1.1/) and IEDB MHC-I processing (https://tools.iedb.org/processing). The parameters in both servers were set to their default values. Peptides with IC_50_ concentration ≤ 100 nM and proteasomal cleavage and transporter associated with antigen processing (TAP) transport scores ≥ 1.0 were subjected to physicochemical properties analysis followed by a pMHCI immunogenicity evaluation using the Class I Immunogenicity (https://tools.iedb.org/immunogenicity/) tool from IEDB. Peptides scoring > 0.25 were deemed immunogenic and subsequently included as final CTL epitopes [20], provided they were antigenic, nontoxic, and nonallergenic.

### 2.3. HTL Epitope Prediction

The NetMHCIIpan server 4.2 (https://services.healthtech.dtu.dk/services/NetMHCIIpan-4.2/) was used to predict potential peptides exhibiting strong binding affinity (< 1.0) across 21 human leukocyte antigen (HLA) alleles [21]. The physicochemical properties of the predicted 15-mer peptides were evaluated. Promiscuous peptides demonstrating antigenicity, nontoxicity, and nonallergenicity were subjected to further immunogenicity testing using the IEDB CD4 immunogenicity prediction server (https://tools.iedb.org/CD4episcore/) [22]. Peptides with a combined score > 45 were deemed immunogenic and categorized as HTL epitopes. Following the identification of the final HTL epitopes, their cytokine-inducing capabilities were assessed. Peptides inducing interleukin-4 (IL-4) and interleukin-10 (IL-10) were identified via IL4Pred (https://crdd.osdd.net/raghava/il4pred/) and IL10Pred (https://webs.iiitd.edu.in/raghava/il10pred/index.html), respectively.

### 2.4. LBL Epitope Prediction

BepiPred (https://tools.iedb.org/bcell/) was used as the main prediction tool for the LBL epitopes. The predicted epitopes were then verified using BcePred (https://crdd.osdd.net/raghava/bcepred/), ABCpred (https://webs.iiitd.edu.in/raghava/abcpred/), and SVMTriP (https://sysbio.unl.edu/SVMTriP/) to avoid server bias [23–26]. Epitopes predicted by BepiPred and at least one of the other three servers were then tested for their physicochemical properties. LBL epitopes that were antigenic, nontoxic, and nonallergenic were considered for the final vaccine construct.

### 2.5. Physicochemical Properties Assessment

Physicochemical evaluations of the predicted epitopes included antigenicity, toxicity, and allergenicity. Antigenic epitopes were identified using VaxiJen v2.0 (https://www.ddg-pharmfac.net/vaxijen/VaxiJen/VaxiJen.html) with the threshold set at ≥ 0.4. All identified antigenic peptides were then assessed for toxicity using ToxinPred (https://crdd.osdd.net/raghava/toxinpred/). Antigenic and nontoxic epitopes were then subjected to allergenicity tests using AllerTOP v.2 (https://www.ddg-pharmfac.net/AllerTOP/) [27]. CTL, HTL, and LBL epitopes that are antigenic, nontoxic, and nonallergenic were included in the final vaccine construct.

### 2.6. Vaccine Construction

The final predicted HTL, CTL, and LBL epitopes were joined using “GPGPG,” “AAY,” and “KK” linkers, respectively. The three epitope groups were then linked together using the “HEYGAEALERAG” linker. The Toll-like receptor 4 (TLR4) agonist 50S ribosomal L7/L12 protein (UniProt ID: P9WHE3) was used as the adjuvant to improve the overall immunogenicity of the vaccine construct. To further optimize the adjuvant by identifying the highly potential immunomodulatory regions, VaxinPAD (https://webs.iiitd.edu.in/raghava/vaxinpad/protein.php) [28] was used. The predicted immunomodulatory peptide with the highest SVM score was appended to the N-terminal of the vaccine construct using the “EAAAK” linker.

The overall mRNA construct starts with a 7-methylguanosine (m^7^G) cap with a triphosphate connected to the first nucleotide (m^7^GpppN), while the 3′ end is a 100-nt poly(A) tail. The cap and the tail aim to increase the stability of the construct. 5′ and 3′ untranslated regions (UTRs) containing regulatory elements of RNA were also added to increase translation efficiency. The ORF is flanked by the 5′ UTR of the adenovirus tripartite leader (TPL) sequence and two *β*-globin 3′ UTRs in a head-to-tail orientation. Between the 5′ cap and the 5′ UTR, a 12-nt poly(A) tail was added to prevent the formation of secondary structures at the 5′ UTR end. The Kozak sequence (GCCACCAUG) containing the start codon was added upstream of the 5′ UTR. Two signal peptides, swine tissue plasminogen activator (tPA) (UniProt ID: Q8SQ23) and swine MHC-I-targeting domain (MITD) (UniProt ID: A0A8D1WGI0), were incorporated into the mRNA vaccine design to increase antigen presentation.

### 2.7. Codon Optimization and Transcription

The translated peptide form composed of the adjuvant and linked epitopes was uploaded to four different codon optimization servers using default parameters: (1) VectorBuilder (https://en.vectorbuilder.com/), (2) GenSmart™ Codon Optimization (https://www.genscript.com/gensmart-free-gene-codon-optimization.html), (3) Java Codon Adaptation Tool (JCat) (https://www.jcat.de/), and (4) Codon Optimization Tool (ExpOptimizer) (https://www.novoprolabs.com/tools/codon-optimization). The sequences generated from each server were uploaded to the GenRCA Rare Codon Analysis Tool (https://www.genscript.com/tools/rare-codon-analysis) to select the most optimized sequence. The most optimized sequence should have a codon adaptation index (CAI) > 0.5, a high tRNA adaptation index (tAI), an effective number of codons (ENC) < 35, and a relative synonymous codon usage (RCSU) > 1. The nucleotide sequence was uploaded to the DNA sequence > RNA sequence > Protein sequence server (https://biomodel.uah.es/en/lab/cybertory/analysis/trans.htm) to be transcribed into mRNA.

### 2.8. Structure Prediction and Evaluation

#### 2.8.1. mRNA Modeling

Using the mRNA sequence, the tertiary structure of the mRNA was predicted using trRosettaRNA (https://yanglab.qd.sdu.edu.cn/trRosettaRNA/), and the secondary structure was predicted by SPOT-RNA integrated into the server. The PDB file of the top-predicted structure was downloaded for molecular docking and pocket binding analysis.

#### 2.8.2. Peptide Modeling

Using the protein sequence, the tertiary structure of the peptide form of the vaccine was predicted using AlphaFold 2 in ColabFold v1.5.5 (https://colab.research.google.com/github/sokrypton/ColabFold/blob/main/AlphaFold2.ipynb), while the secondary structure was characterized using GOR4 (https://npsa-prabi.ibcp.fr/cgi-bin/npsa_automat.pl?page=/NPSA/npsa_gor4.html). The top-predicted tertiary structure was then refined using GalaxyRefine (https://galaxy.seoklab.org/cgi-bin/submit.cgi?type=REFINE) in the GalaxyWEB server. The quality of the top-refined structure was evaluated using protein structural analysis (ProSA) (https://prosa.services.came.sbg.ac.at/) [29] and ERRAT and PROCHECK in SAVES v6.0 (https://saves.mbi.ucla.edu/) [30]. The structure was considered to be of good quality if it fulfilled the following criteria: (1) *z*-score between −10 and 10 in ProSA, (2) overall quality factor of at least 95% in ERRAT, and (3) at least 90% residues in the most favored regions in the PROCHECK Ramachandran plot. ChimeraX was used to visualize the final structure.

### 2.9. Molecular Docking and Analysis

#### 2.9.1. Protein–Protein Docking and Molecular Dynamics

For protein–protein docking, the refined final peptide model was uploaded to ClusPro 2.0 (https://cluspro.bu.edu/login.php?redir=/queue.php) and docked to TLR4. The binding energy of the top cluster was evaluated using PRODIGY (https://wenmr.science.uu.nl/prodigy/), and the interactions between the vaccine construct and TLR4 were assessed using PDBsum (https://www.ebi.ac.uk/thorntonsrv/databases/pdbsum/Generate.html). TLR4-agonist complexes, such as TLR4–*Brucella* lumazine synthase, TLR4–*M. tuberculosis* RpfE, TLR4–*Streptococcus pneumoniae* DnaJ, and a previously designed multiepitope ASF vaccine–TLR4 were used as controls for the binding affinity assessments [16]. Furthermore, the stability of the TLR4–vaccine construct was assessed via coarse-grained (CG) molecular dynamics simulations in GROMACS 2023.2, employing the SIRAH force field and explicit solvent WatFour (WT4) [31]. Two energy minimization processes were executed: (1) side chain energy minimization with backbone restraint and (2) full system energy minimization. Each minimization step comprised 50,000 steps utilizing the steepest descent algorithm. Subsequently, the system was equilibrated for 5000 ps at a temperature of 300 K and pressure of 1 bar. The root-mean-square deviation (RMSD) was calculated during the production simulation, which extended over 100 ns.

Atomistic molecular dynamics was also performed in GROMACS 2023.3 employing the CHARMM36 forcefield and TIP3P water model. Energy minimization was performed for 50,000 steps using the steepest descent algorithm. The remaining steps followed the parameters during the CG molecular dynamics simulation. In addition to the RMSD, the root-mean-square fluctuation (RMSF), radius of gyration (Rg), number of hydrogen bonds, and solvent-accessible surface area (SASA) were determined.

#### 2.9.2. Protein–RNA Docking and Binding Pocket Dynamics Analysis

The mRNA form of the vaccine construct was docked to TLR3 and TLR7 using the HDOCK server (https://hdock.phys.hust.edu.cn/) with all the parameters set to default. The binding model with the most negative docking score was downloaded for binding pocket dynamics analysis using D3Pockets (https://www.d3pharma.com/D3Pocket/) and then viewed using the PyMol software.

### 2.10. Immune Simulation

**Figure 1:**
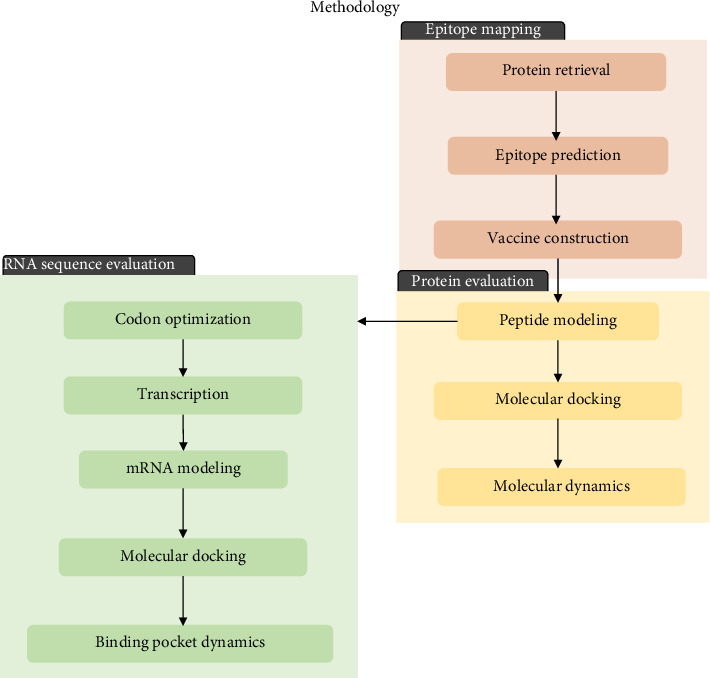
Overview of the methodology for designing an mRNA vaccine against ASF composed of three major steps: epitope mapping, evaluation of the protein form of the vaccine construct, and evaluation of the mRNA form of the vaccine construct.

The immune response profile of the completed vaccine design was assessed using the online immune simulation platform C-ImmSim (https://kraken.iac.rm.cnr.it/C-IMMSIM/index.php?page=1). Three injections were administered at 4-week intervals. The time steps were configured at intervals of 1, 84, and 168 with a random seed value of 12,345. The simulation volume was set to 50, and the number of simulation steps was set to 1000. All other parameters were maintained at default settings. The summary of the methodology utilized in this study is shown in Figure 1.

## 3. Results and Discussion

ASFV remains a serious threat to the swine industry. However, current vaccine development efforts are inadequate in terms of the safety of live-attenuated vaccines and the effectiveness of whole-inactivated and current subunit vaccines against ASF [32, 33] prompting the need to discover novel antigens and vaccine types. RV is a genome-based approach that has transformed the vaccine development field by allowing the identification of antigens without the need to culture the pathogen, regardless of its purified quantity requirement for vaccine testing [34, 35]. Several studies have established the efficacy of RV-designed vaccines including the multiepitope vaccine by Liang et al. [36] against *Streptococcus suis* in pigs. In vitro validation of the vaccine in mice models showed a successful induction of protective effects against *S. suis* and reduction of histopathological damage. Another successful vaccine was developed against *Mycoplasma hyopneumoniae* (Mhp) causing *Mycoplasma* pneumonia in swine (MPS). Li and colleagues [37] were able to design a vaccine that induced humoral and cellular immune responses in immunized mice and piglets. Aside from veterinary use, RV has also been used to design vaccines intended for human use. Jiang et al. [38] designed a multiepitope vaccine against *Mycobacterium* tuberculosis. In vitro validation of the designed vaccine showed elevated IFN-y + T lymphocytes and production of IFN-y, TNF-am IL-6, and IL-10 cytokines in active tuberculosis patients.

RV coupled with bioinformatics improves the efficiency of the production process and stability of mRNA vaccines by focusing only on predicting vital epitopes [15]. RV allows for the efficient discovery of antigens using genomic information, which is especially advantageous for the large and complex genomes of ASFV [39]. The structural proteins p30, p54, p72, and p22 have been identified by several studies to be dominant antigens of ASFV [40–43]. Other unassigned and nonstructural proteins, such as pB475L, pE184L, and pK145R, have also been reported to induce antibody development in ASFV infection [43]. Considering the size of the ASFV genome, more unidentified determinants might be involved in the protection against ASFV challenge highlighting the importance of a pan-proteomic approach in vaccine design.

### 3.1. Proteome Retrieval and Conservancy Analysis

A pan-proteomic approach was used to identify potential vaccine antigens in the ASFV genome. A total of 197 distinct ASFV proteomes were retrieved from the NCBI and UniProt databases. After excluding 21 proteomes containing fewer proteins than the standard count of 150, six proteomes with incomplete genome coverage, and 70 proteomes with either unverified tags from NCBI or from the excluded section of UniProt, only 100 well-annotated ASFV proteomes remained. Among these, 33 proteomes had genotype assignments available from NCBI and were used as reference sequences to determine the genotype of the remaining 67 proteomes through phylogenetic-based sequence clustering. The 100 proteomes were distributed into 11 genotypes, with the isolates predominantly belonging to Genotype II, followed by Genotype I. These two genotypes have been associated with outbreaks in Africa and are the only genotypes associated with outbreaks outside Africa [3, 4]. ASFV Genotype I was responsible for the first case of ASF reported in Asia. On the other hand, ASFV Genotype II, the most prevalent genotype in Southeast Asia, has been responsible for the loss of over seven million swine in Eurasian countries since its initial spread to China and other Asian countries in 2018 [1, 10, 44]. Therefore, this study focused on the proteins found in Genotypes I and II.

The final ASFV proteomes comprised 18,858 proteins, 2978 of which were identified as nonredundant by CD-HIT. Among all nonredundant proteins, only 163 were found in at least 80% of Genotype I or Genotype II proteomes. From these 163 protein sequences, 93.80% of the amino acid residues were identified as conserved by the PVS. 532 peptides containing 9 consecutively conserved amino acids were selected for the prediction of CTL epitopes, while 450 peptides with 15 consecutively conserved amino acids were chosen for HTL epitope prediction. Since a larger genome size may lead to an increase in the genetic diversity, ensuring the conservancy of the epitopes to be incorporated into the vaccine design increases its cross-protection potential against a wide range of Genotype I and II variants.

### 3.2. Epitope Prediction

An effective vaccine should be able to trigger the formation of immunological memory within the adaptive immune system. The adaptive immune system comprises two key responses: the humoral immune response, involving antibodies produced by B lymphocytes, and the cellular immune response, mediated by T lymphocytes [45, 46]. This necessitates the integration of CTL, HTL, and LBL epitopes into the vaccine design. By incorporating these epitopes, the vaccine aims to provoke a comprehensive immune response, encompassing both antibody-mediated defense and cellular immunity.

In cell-mediated responses, there are two classes of T lymphocytes: cytotoxic T cells or CD8^+^ T cells and helper T cells or CD4^+^ T cells. The role of T-cell immunity in providing protection against ASFV has already been proven in various studies [47–50], especially for CD8^+^ T cells [41]. Given the roles of both CD8^+^ and CD4^+^ T cells, the induction of both classes is important during ASFV infection.

Activated cytotoxic T cells offer protection against intracellular pathogens that multiply in the cytoplasm using cytotoxic molecules, such as the Fas ligand (FasL), perforins and granzymes, or TNF-related apoptosis-inducing ligand (TRAIL) [49, 51–53]. During ASFV infection, the effector activity of CTLs is mainly attributed to death receptor-mediated apoptosis by FasL or TRAIL, rather than direct lysis by perforin [49]. Two of the most important components of cytotoxic T cells are T-cell receptors (TCRs) on the cell surface and lytic granules in the cytoplasm. Cytotoxic T-cell activation relies on the recognition of antigens bound to MHC Class I by a TCR and is thus the main basis for the prediction of CD8^+^ T-cell epitopes [54, 55]. This study predicted 47,165 potential CTL epitopes from 532 peptides with ≥ 9 consecutive conserved amino acids, based on their binding affinity to at least nine swine leukocyte antigens (SLA). These putative CTL epitopes undergo further filtering following the MHC Class I antigen presentation pathway to ensure successful antigen presentation.

The MHC Class I antigen presentation pathway includes proteolytic processing of proteins in the cytosol by the proteasome. The processed proteins are then translocated from the cytosol into the ER lumen by the TAP and bind to MHC Class I facilitated by tapasin. Peptide-loaded MHC Class I molecules are transported to the plasma membrane [54, 56]. Thus, aside from the binding affinity of peptides to MHC-I alleles, the final nonallergenic, nontoxic, and antigenic CTL epitopes should have an immunogenicity score > 0.25, C-terminal cleavage affinity, and TAP efficiency score ≥ 1.0. Six CTL epitopes fulfilled all the criteria described previously and are listed in Table 1.

On the other hand, activated HTLs provide defense against both extracellular and intracellular pathogens. The effector HTL aids in the stimulation of B cells to produce antibodies for the inactivation or elimination of extracellular pathogens. HTLs target intracellular pathogens by activating macrophages and CTLs [54]. Studies have demonstrated that CD4^+^ Th cells play a role in the effective generation of antibodies and T cell–dependent class switches during ASFV infection [49, 57, 58]. Although the activation of naive CD4^+^ T cells into effector cells also relies on the recognition of TCR by the peptide bound to MHC Class II molecules, MHC Class II molecules can accommodate larger peptides due to their relatively bigger groove. Proteins are processed through the endosomal pathway before binding to MHC Class II molecules and presented on the cell surface [59]. Of the 450 peptides with ≥ 15 consecutively conserved amino acids, 44,773 putative HTL epitopes were predicted. Five final HTL epitopes were identified as nonallergenic, nontoxic, and antigenic, with > 20 immunogenicity scores and strong binding affinity to four HLAs.

Effector CD4^+^ cells differentiate into Th1 and Th2 cells. Th1 cells produce Type I cytokines, such as IFN-*γ* and IL-10, whereas Th2 cells produce Type II cytokines, including IL-4 and IL-5. Th17 cells release IL-17, which plays a crucial role in attracting phagocytic cells to the sites of inflammation. Th1 cells play a vital role in combating intracellular pathogens by enhancing cell-mediated responses and activating macrophages, while Th2 cells are significant during parasitic infections as they support the humoral immune response. Th17 cells are important for infections caused by extracellular bacteria [51, 60]. Furthermore, effector CD4^+^ cells stimulate the T cell–dependent aspect of humoral immunity, wherein CD4^+^ cells recognize antigens that typically evoke weak or absent B-cell responses [53]. Two servers were used to predict the IL-4- and IL-10-inducing ability of the final HTL epitopes (Table 2). Two HTL epitopes were predicted to stimulate the production of both IL-4 and IL-10 while two other epitopes were found to induce either IL-4 or IL-10.

**Figure 2:**
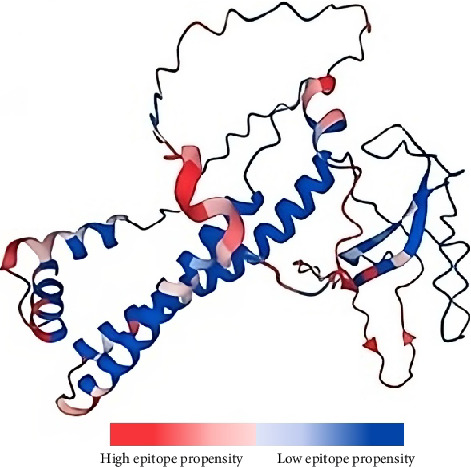
Predicted conformational B-cell epitopes in the predicted tertiary structure of the vaccine design. A deeper red color indicates a higher epitope propensity, while a deeper blue color indicates lower epitope propensity.

In addition to the cellular response, the humoral response is a crucial aspect of the adaptive immune system. Specifically, antigen–antibody interactions are vital for triggering a humoral response against invading pathogens. Antibodies play various roles, including neutralizing infectivity, facilitating phagocytosis, initiating antibody-dependent cellular cytotoxicity (ADCC), and mediating complement-mediated lysis of pathogens or infected cells [61]. Antibodies recognize specific regions of an antigen known as B-cell epitopes [62]. B-cell epitopes can be either continuous stretches of amino acids, known as linear epitopes, or amino residues brought together through protein folding, also referred to as conformational epitopes [63]. Unlike T cells, B cells recognize intact antigens [51]. Since no peptide processing is needed before antibody recognition, the prediction of B-cell epitopes is relatively straightforward. Additionally, the optimal length for maximum activity of B-cell epitopes has not been identified; hence, multiple servers are usually utilized to improve accuracy [64–66]. This study identified four linear nonallergenic, nontoxic, and antigenic B-cell epitopes predicted by at least two servers, as listed in Table 3. In addition, 12 residues across ten B-cell epitopes, with propensity values ranging from 1.58957 to 2.8465, were predicted (Figure 2).

Although the association between the presence of neutralizing antibodies and protection has been previously demonstrated [64], the anti-ASFV antibody is not enough to protect pigs against ASFV [48, 49], further highlighting the importance of inducing both cellular-mediated and humoral immune responses for successful protection during ASFV infection.

### 3.3. ASFV mRNA Vaccine Assembly

Stabilization of mRNA is associated with high expression, since mRNA is prone to degradation. The stability and translation efficiency of mRNA are directly attributed to its structural characteristics, such as the 5′ cap, poly(A) tail, and UTRs [67]. To protect mature mRNA from degradation, 5′ capping was required. This facilitates the recruitment of ribosomes, gene expression, and self- versus non-self-identification [68]. The m^7^G binds to 5′-5′ triphosphate (m^7^GpppN) to form a cap structure. The cap facilitates the binding of eukaryotic translation initiation factor 4E (*eIF4E*) to initiate transcription [69]. At the 3′ end, a 100-nt poly(A) tail was added to prevent mRNA degradation and promote translation [70]. Tail size affects mRNA decay through modulation of 3′ exonucleolytic degradation. This study incorporates a 100-nt poly(A) tail that has been previously determined to be ideal [67, 71]. A 12-nt poly(A) tail was also added between the 5′ cap and 5′-UTR to avoid the formation of a secondary structure at the 5′ end of the construct, thus increasing protein translation [72, 73].

The 5′- and 3′-UTRs are also known to regulate mRNA stability and protein translation. Specifically, the 5′-UTR is essential for the recruitment of ribosomes and represents the site where the preinitiation complex of protein translation forms, while the 3′-UTR is associated with the stabilization of intracellular mRNA [68]. Additionally, these UTRs can be further adjusted to increase stability and translation accuracy. An effective 5′-UTR should: (i) avoid the presence of start codons and noncanonical start codons within the 5′-UTR as they disrupt ORF translation; (ii) prevent ribosome recruitment and codon recognition by avoiding highly stable secondary structures; and (iii) be short, which previous research has shown to be more conducive to mRNA translation [74]. This study also used the TPL previously proven by Reshetnikov et al. [75] to have a high translation efficiency when used as the 5′-UTR, while Holtkamp et al. [76] demonstrated that two reiterated *β*-globin 3′-UTRs showed a significant increase in protein levels and prolonged persistence of protein. The Kozak sequence was also added, which contains the start codon (AUG) to initiate the translation process.

**Figure 3:**
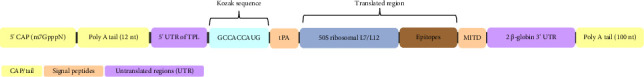
The final assembled mRNA vaccine composed of the adjuvant, predicted epitopes, 5′ cap, poly(A) tail, UTRs, Kozak sequence, and signal peptides.

In addition to structural components, signal peptides were incorporated to increase the translation efficiency. The tPA directs the target protein from the cell, while the MITD directs CTL epitopes to the MHC-I compartment of the endoplasmic reticulum [77]. Since these peptides are expected to be cleaved while entering the secretory pathway and MHC-I pathway [78], the translated peptide form of the mRNA vaccine was used to predict the different properties of the vaccine construct. The final mRNA vaccine with all the aforementioned components was assembled as shown in Figure 3.

### 3.4. Translated Region of the mRNA Vaccine

The translated peptide form of the mRNA vaccine is composed of the predicted epitopes and adjuvant. The final six CTL epitopes, five HTL epitopes, and four LBL epitopes were connected using “AAY,” “GPGPG,” and “KK” linkers, respectively. The “AAY” (Ala-Ala-Tyr) linker is a proteosome cleavage site in mammalian cells that effectively separates epitopes within the cells, while the “GPGPG” linker used to link the HTL epitopes is a universal spacer that was demonstrated to induce HTL responses. “KK,” a lysine linker that links B-cell epitopes, is a target of cathepsin B, which is a lysosomal protease for the processing of peptides to be presented on the cell surface in an MHC-II-restricted antigen presentation. These three linkers mainly ensure the retention of immunogenicity of the individual epitopes by reducing junctional immunogenicity [79, 80]. The “HEYGAEALERAG” linker was used to connect the different epitope groups to each other. This linker has five cleavage sites that are important for proteasomal and lysosomal degradation [80].

**Figure 4:**
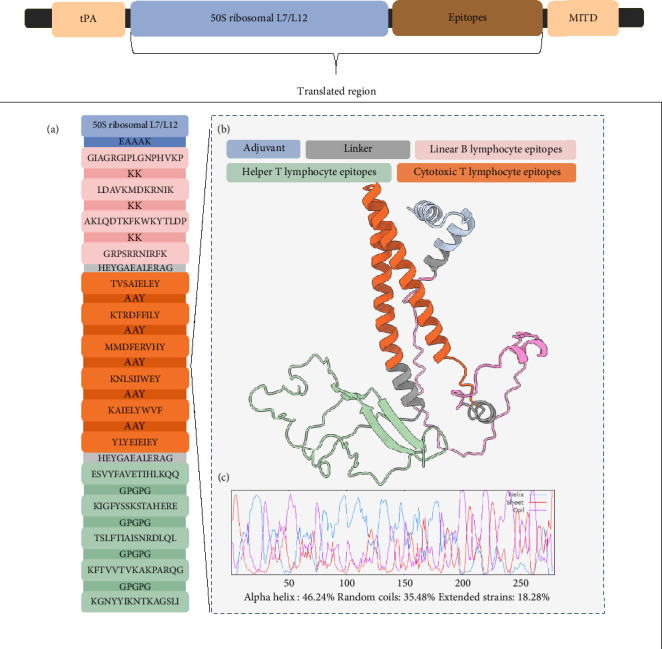
Assembled adjuvant and predicted epitopes of the (a and b) translated region of the final vaccine construct, and (c) its predicted secondary structure.

Epitope-based vaccines usually confer limited immunity; hence, adjuvants are typically added to increase immunogenicity [81–83]. This study used a TLR4 agonist, 50S ribosomal protein L7/L12, as the adjuvant for the final vaccine design. To optimize the adjuvant, VaxinPAD was used to predict its potential immunomodulatory regions. VaxinPAD uses two datasets containing experimentally validated immunomodulatory peptides from the literature to serve as a positive set and endogenously circulating human peptides to serve as a negative set. A support vector machine (SVM)–based computational model then classifies query peptides as A-cell epitope (positive) or nonepitope (negative) [28]. From the 130 residues comprising the adjuvant, a 25-mer region that was identified to be immunomodulatory was linked to the linked epitopes using the “EAAAK” linker, which is a rigid linker that separates the adjuvant from the rest of the epitopes with minimal interference, thereby retaining the separate functional properties of the adjuvant and the rest of the vaccine [80]. This formed the translated region of the vaccine construct (Figure 4). The translated region of the vaccine construct is 279 aa long and is predominantly composed of alpha helices (46.24%), followed by random coils (35.48%), and extended strands (18.28%) (Figure 4). Analysis of the physicochemical properties of the construct showed that it was antigenic, nonallergenic, soluble, and stable (Table 4). It has an aliphatic index of 81.22 and a GRAVY value of −0.425. A high aliphatic index demonstrates its stability at a wide temperature range, attributable to the large volume of aliphatic side chains relative to the protein, and a negative GRAVY value indicates hydrophilicity and strong interactions with water molecules [83, 84].

### 3.5. Peptide Tertiary Modeling

**Figure 5:**
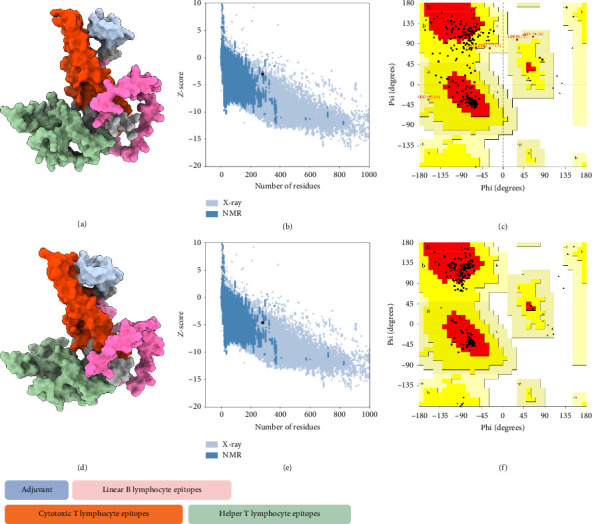
Quality comparison of the unrefined and refined tertiary structure predicted using AlphaFold 2. (a) Raw predicted tertiary structure of the vaccine construct visualized using ChimeraX, (b) a *z*-score value of −3.02 (black dot), and (c) Ramachandran plot showing 71.9% of residues in the most favored region (black dots). (d) Successful refinement of the structure, (e) generated a *z*-score of −4.58 (black dot) with (f) 90.2% of the residues in the most favored region (black dots).

The tertiary structure of the vaccine construct was predicted using AlphaFold 2 for molecular recognition [85, 86]. AlphaFold 2 topped the CASP 14 ranking by a significant margin [87]. The top-predicted unrelaxed model was downloaded for quality assessment. The predicted model had an ERRAT value of 84.43% and a *z*-score of −3.02, with 71.9% of the residues found in the most favored regions in the Ramachandran plot. To improve the quality of the predicted structure, structural refinement was performed. The refined structure had an improved ERRAT score of 95.31% and a *z*-score of −4.58, with 90.2% of the residues found in the most favored regions of the Ramachandran plot and 9.8% in additional allowed regions, indicating a successful refinement (Figure 5).

### 3.6. Molecular Docking and Molecular Dynamics

**Figure 6:**
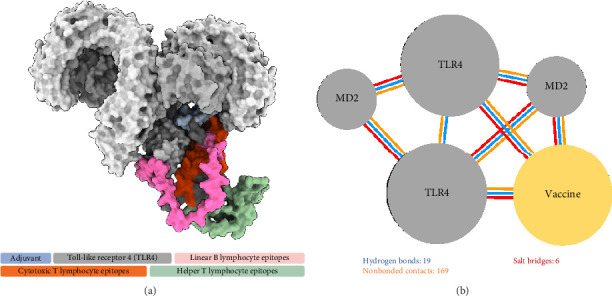
Molecular docking of the vaccine construct with TLR4. (a) Top cluster predicted by ClusPro of the docking of TLR4 (grey) with the vaccine construct (colored) visualized using ChimeraX and (b) the predicted molecular interactions by PDBsum in 2D.

The refined tertiary structure was docked to TLR4 using ClusPro. TLR4 is a protein-recognizing TLR that plays a key role in the innate immune response. Binding of the construct to TLR4 upregulates proinflammatory cytokines through the activation of transcription factors via MyD88-dependent and MyD88-independent pathways [88]. TLRs also bridge the innate and adaptive immune responses. During TLR signaling in innate immune cells, DC maturation and regulatory cytokine production are regulated, which indirectly regulates T-cell differentiation and proliferation [89, 90], thereby strengthening the adaptive immune response for the generation of antigen-specific memory cells. 50S ribosomal protein L7/L12, specifically, stimulates the innate immune response through TLR4 recognition. This event induces the maturation of DCs as well as the production of proinflammatory cytokines. The DCs then activate naïve T cells and induce T cell–mediated cytotoxicity [91]. Several studies incorporated 50S L7/L12 protein in their vaccine design as adjuvant for use in swine [92, 93], fish [94], and humans [95, 96] targeting different pathogens highlighting its capability to elicit immune response in a wide range of host. TLR4–vaccine docked complex (Figure 6(a)) exhibited a good binding affinity characterized by a binding free energy (Δ*G*) of −14.7 kcal/mol, which is higher than all of the controls used in this study, including TLR4–*Brucella* lumazine synthase, TLR4–*M. tuberculosis* RpfE, TLR4–*S. pneumoniae* DnaJ, and a previously designed multiepitope ASF vaccine–TLR4 with binding energies of −9.9, −9.7, −13.1, and −14.0 kcal/mol.

Additionally, 19 hydrogen bonds, six salt bridges, and 169 nonbonded contacts were identified between the vaccine construct and TLR4 (Figure 6(b)). Hydrogen bonding is an essential interaction for the stabilization of protein–ligand complexes. More hydrogen bonds indicate more stable binding [97]. Similarly, salt bridges contribute to protein–protein stability [98]. TLR4–vaccine binding had the highest number of hydrogen bonds compared with the controls further demonstrating successful binding.

To further test the stability of the TLR4–vaccine complex, 100-ns atomistic and CG molecular dynamics simulations were performed. After simulation, RMSD, RMSF, Rg, and SASA analyses were calculated to assess stability.

**Figure 7:**
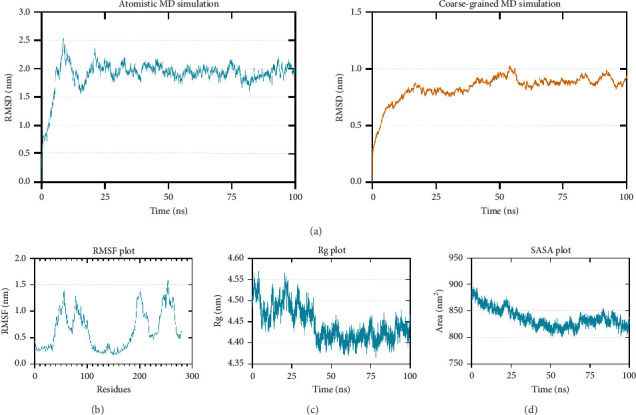
100-ns molecular dynamics simulation for assessing the stability of the vaccine when docked to TLR4. (a-left) Atomistic and (a-right) coarse-grained RMSD, (b) RMSF, (c) Rg, and (d) SASA analyses show that the TLR4–vaccine complex is stable.

RMSD calculation is used to measure the difference between the initial and final structural conformations of a protein. Deviations during the course of the simulation are related to protein stability [99, 100]. A stable protein structure generates smaller deviations; hence, a plateau in the RMSD graph indicates stabilization. The calculated RMSD for both the atomistic (Figure 7(a) left) and CG (Figure 7(a) right) molecular dynamics simulations shows that the TLR4–vaccine docked complex stabilized at approximately 25 ns.

While RMSD measures the conformational difference of the whole protein, RMSF measures the fluctuation of individual amino acid residues [101]. Higher RMSF values signify flexible regions, while lower RMSF values imply restricted movements, indicating stability [102, 103]. The lowest RMSF values were found in the adjuvant region of the vaccine construct (residues 0–33) and the T-cell epitope region (residues 111–261) which can be attributed to the agonistic property of the 50S ribosomal protein L7/L12 as previously discussed (Figure 7(b)).

Rg is related to the compactness of the protein. It measures the conformational changes and flexibility of a protein over time [104, 105]. Figure 7(c) shows that the Rg-time fluctuations were within 4.35 to 4.60 nm. Lastly, SASA predicts the area of the protein exposed to the surrounding solvent molecules for interaction. Increasing SASA values during simulation indicate protein relaxation and, consequently, protein instability [104]. Figure 7(d) shows a decreasing trend in the SASA values, which is supported by the Rg plot indicating an increase in stability.

**Figure 8:**
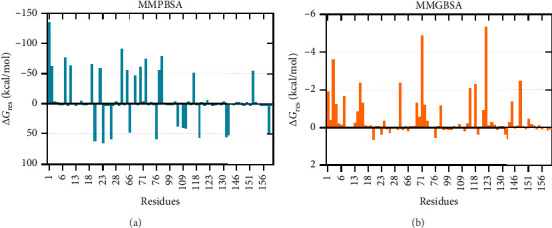
Per-residue energy decomposition of the ligand (vaccine construct) calculated using (a) MMPBSA and (b) MMGBSA.

The binding free energies of the complex were determined through molecular mechanics generalized born surface area (MMGBSA) and molecular mechanics Poisson–Boltzmann surface area (MMPBSA) methods, together with per-residue energy decomposition [105, 106]. The binding energies calculated using MMGBSA and MMPBSA were −73.74 kcal/mol and −133.58 kcal/mol, respectively. The energy contribution of each interface residue in the ligand was determined by energy decomposition analysis. Fifty-three contact residues were identified by MMG(P)BSA analyses to be the highest contributors to the binding affinity (Figure 8).

### 3.7. Codon Optimization and Transcription

In addition to stability, another main goal in mRNA vaccine design is codon optimality [10]. Codon optimization usually involves altering rare codons in the sequence to preferred synonymous codons without changing the amino acid sequence [67], since the use of synonymous codons varies depending on the abundance of transfer RNAs (tRNAs) in cells. An optimized vaccine mRNA sequence aims to improve translation elongation efficiency to decrease the number of vaccine mRNA copies required [107]. Four different servers were used for codon optimization: VectorBuilder, GenSmart Codon Optimization, JCat, and ExpOptimizer. Several indices have been introduced to measure codon optimality or translational fitness (TF) [74]. In this study, the optimized sequence of vaccine construct from each server was compared in terms of CAI, tAI, ENC, and RCSU using the GenRCA Rare Codon Analysis Tool.

CAI and tAI were previously identified as appropriate indices for measuring the deviations in the coding regions of the sequence from uniform codon usage. CAI measures the extent to which the coding sequence represents the usage of codons in an organism and is the primary index used to predict gene expression with a CAI value > 0.5, indicating high gene expression levels [108, 109]. tAI values estimate the gene-level translation efficiency because the tAI of each codon is proportional to the tRNA that can translate this codon [110]. A high tAI in mRNA is also associated with a long half-life [111].

As with the two aforementioned indices, ENC and RCSU only need the coding sequences to compute the deviation from the “uniform” or expected “background” distribution [112]. An ENC value < 35 indicates a strong codon bias, with a decreasing ENC value that is associated with an increasing codon usage bias (CUB). A high CUB is found in highly expressed genes [113]. An RSCU value > 1 indicates positive codon bias and stronger host adaptability [114]. Among the four optimized sequences, JCat had the most optimized sequence in terms of the four indices mentioned previously (Table 5). The optimized nucleotide sequence will be subjected to in silico transcription to obtain the RNA sequence for tertiary structure modeling and docking.

### 3.8. Predicted Tertiary Structure of the mRNA Form of the Vaccine Construct

**Figure 9:**
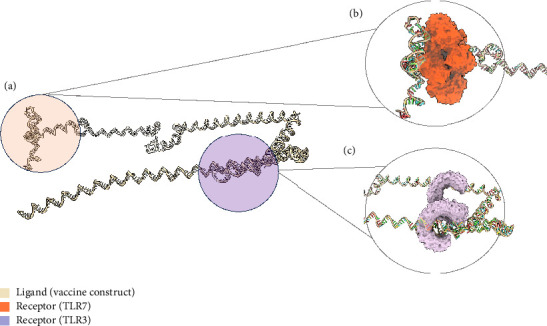
Interaction of RNA-recognizing TLRs and the mRNA form of the vaccine. (a) Predicted tertiary structure of the mRNA form of the vaccine docked to (b) TLR 7 and (c) TLR3.

The tertiary structure of the mRNA vaccine was predicted using trRosettaRNA (Figure 9(a)). This server combines the methods of AlphaFold 2 and trRosetta [115]. In CASP15, trRosettaRNA outperformed other deep learning–based methods [116]. The top-predicted structure was docked to TLR3 and TLR7 using HDOCK (Figures 9(b) and 9(c)). HDOCK allows for protein–RNA/DNA docking server and has been frequently used by biologists in their research [117]. The stability of the docked complexes was then evaluated through binding pocket analysis.

### 3.9. Binding Pocket Dynamics

mRNA acts as both a guide for the generation of immunogens and as adjuvant. Upon entering the cells, mRNAs can be sensed by intracellular innate RNA sensors, consequently activating the innate immune response. Endosomal TLRs, such as TLR3 and TLR7, are the pathogen recognition receptors (PRRs) of viral RNA. These TLRs can recognize single-stranded RNA (TLR7) or double-stranded RNA (TLR3). Generally, activation of multiple innate immune responses is preferred to initiate and strengthen the adaptive immune response; however, activation of the innate immune sensor leads to uncontrolled local and systemic inflammation and low translation efficiency due to downregulation of antigen expression from mRNA [118]. Additionally, the activation of PRRs leads to the secretion of proinflammatory cytokines, which decreases protein expression and secretion [119]. Since the efficacy of an mRNA vaccine is dependent on its translation efficiency [73], a docking analysis of the mRNA form of the vaccine was performed. The vaccine construct was docked to endosomal TLRs 3 and 7. The binding stability of the TLR–vaccine complex was evaluated through binding pocket analysis using D3Pockets. D3Pockets is an online web server that uses MD simulation trajectories or conformational assemblies to identify binding pocket regions in a .pdb file and determine the dynamic properties of possible ligand-binding pockets [120].

**Figure 10:**
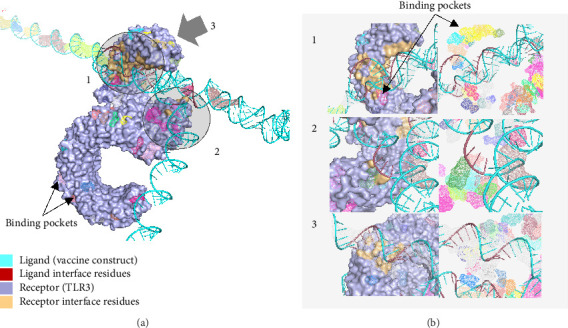
The mRNA form of the vaccine docked to TLR3. (a) Binding pocket analysis of the construct to TLR3 indicated that only one contact region has a binding pocket, implying a high translation efficiency of the designed mRNA vaccine. (b) The identified contact regions between the vaccine construct and TLR3.

**Figure 11:**
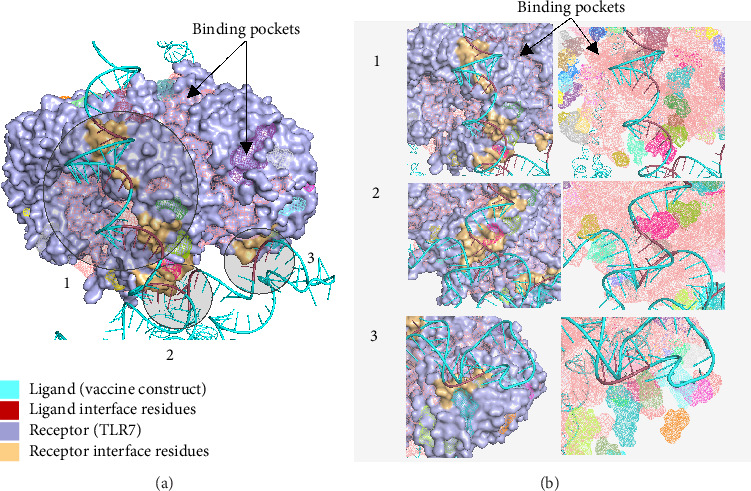
The mRNA form of the vaccine docked to TLR7. (a) Binding pocket analysis of the construct to TLR7 indicates that the no binding pockets were found in the contact regions occurring implying high translation efficiency of the designed mRNA vaccine. (b) The identified contact regions between the vaccine construct and TLR7.

Figure 10(a) shows the binding pocket analysis of the vaccine construct bound to TLR3. Three contact regions (1–3) were identified with the contact residues of the receptor and the ligand (vaccine) highlighted in orange and red, respectively. Only one binding pocket (Figure 10(b)-3) was found in the contact regions of the TLR3 and vaccine construct indicating minimal interaction between the two (Figure 10(b)). Similarly, the vaccine construct bound to TLR7 showed three contact regions with no binding pockets (Figure 11) suggesting no interactions. Overall, the vaccine construct can avoid the detection of innate sensors indicating a potentially high translation efficiency.

### 3.10. Immune Simulation

**Figure 12:**
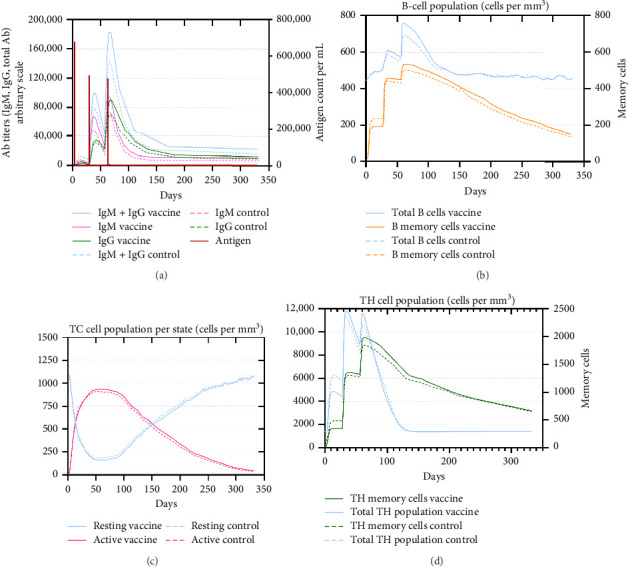
Immune simulation after immunization generated by C-ImmSim. (a) Antibody titer population, (b) B-cell population, (c) TC cell population, (d) TH cell population.

An immune simulation was performed to evaluate the immune response of the host postimmunization. There was a significant increase in the total antibody titers after the third immune response even after antigen reduction. This suggests the potential generation of immune memory and practiced immunity (Figures 12(a) and 12(b)). Immunoglobulin M (IgM) levels were higher than the immunoglobulin G (IgG) levels suggesting induction of primary response after antigen exposure [121], which implies effective humoral response. There was also an increase in the B-cell population, including memory B cells, indicating memory formation and isotype switching (Figure 12(b)). The memory helper cells remained high for the entire simulation period (Figure 12(c)), similar with the active T cytotoxic cell populations showing elevated levels throughout the simulation (Figure 12(d)). Overall, these results suggest that the multiepitope mRNA vaccine construct elicits a robust and diverse immune response to ASFV.

## 4. Conclusion

In conclusion, the current vaccine development efforts for ASFV are falling short in terms of safety and effectiveness. To address this challenge, there is a need to discover novel antigens through RV, allowing for the identification of antigens without the need to culture the pathogen. Furthermore, with the rapid emergence of recombinant strains [35], rapid or improved vaccine designs have shortened vaccine development. In this study, a pan-proteomic approach was used to identify potential vaccine antigens from the ASFV genome, prioritizing proteins found in Genotypes I and II, which are associated with global outbreaks. Additionally, CTL, HTL, and LBL epitopes were predicted from these proteins to induce both cellular and humoral immune responses.

The final mRNA vaccine construct was designed to incorporate these epitopes, along with other components to enhance stability and translation efficiency. A TLR4 agonist was also selected as an adjuvant to boost immunogenicity. Codon optimization and tertiary structure prediction were performed to ensure the high antigen expression and predict innate immune activation via TLR interactions. Molecular dynamics simulations confirmed the stability of the vaccine construct and its binding to TLR4.

Furthermore, the mRNA form of the vaccine was analyzed to assess its potential to evade innate immune sensors and maintain a high translation efficiency. Immune simulation results have demonstrated that the multiepitope mRNA vaccine construct elicits a robust and diverse immune response, suggesting its potential effectiveness against ASF.

Overall, this study presents a promising approach for developing an effective and safe mRNA vaccine construct that can induce both cellular and humoral immune responses for comprehensive protection against ASF.

## Figures and Tables

**Table 1:** Predicted CTL epitopes and their corresponding antigenicity and immunogenicity values.

CTL epitopes	Protein source	Antigenicity scores	Immunogenicity scores
TVSAIELEY	Polyprotein p220 (CP2475L)	1.71	0.25866
KTRDFFILY	Transmembrane (C62L)	1.49	0.36916
MMDFERVHY	Cysteine protease (S273R)	1.44	0.32496
KNLSIIWEY	MGF 360-13L	1.41	0.33025
KAIELYWVF	MGF 360-18R	1.36	0.34073
YLYEIEIEY	Guanylyltransferase (NP868R)	1.09	0.49422

**Table 2:** Predicted HTL epitopes, their protein sources, and their corresponding physicochemical properties and cytokine-inducing abilities.

HTL epitopes	Protein source	Antigenicity	Immunogenicity	IL-4	IL-10
ESVYFAVETIHLKQQ	EP424R	1.12	49.94	YES	YES
KIGFYSSKSTAHERE	Helicase/primase (F1055L)	1.33	41.19	NO	YES
TSLFTIAISNRDLQL	MGF 505-3R	1.16	39.39	YES	YES
KFTVVTVKAKPARQG	p104R	1.38	44.78	YES	NO
KGNYYIKNTKAGSLI	pF1055L	1.20	49.30	NO	NO

**Table 3:** Predicted LBL epitopes, their protein sources, and their corresponding antigenicity scores.

LBL epitopes	Protein source	Antigenicity scores
GIAGRGIPLGNPHVKP	Minor capsid protein (B438L)	1.43
LDAVKMDKRNIK	Envelope protein p22 (KP177R)	1.03
AKLQDTKFKWKYTLDP	Transmembrane protein (C257L)	1.37
GRPSRRNIRFK	Major capsid protein (B646L)	1.83

**Table 4:** Physicochemical properties of the translated form of the vaccine construct.

Properties	Results
Theoretical pI	9.67
Estimated half-life	1.3 h (*E. coli*)
Instability index (II)	31.18
Aliphatic index	81.22
Grand average of hydropathicity (GRAVY)	−0.425
Antigenicity	0.9711
Solubility	Soluble (58.50%)
Allergenicity	Non-allergen

**Table 5:** CAI, tAI, ENC, and RCSU of the optimized sequences generated by four different servers.

	VectorBuilder	GenSmart	JCat	ExpOptimizer
CAI	0.95	0.74	0.81	0.76
tAI	0.27	0.24	0.29	0.25
ENC	26.09	44.44	20.4	46.93
RCSU	2.54	1.47	3.28	1.45

## Data Availability

The data used to support the findings of this study are included in the article. Additional data can be made available upon request.
